# Tricolored bats at a southern range edge exhibit partial migration northward in autumn

**DOI:** 10.1186/s40462-022-00358-x

**Published:** 2022-12-02

**Authors:** Lisa M. Smith, Jeffery A. Gore, Terry J. Doonan, Caitlin J. Campbell

**Affiliations:** 1 Fish and Wildlife Research Institute, Florida Fish and Wildlife Conservation Commission, 1105 SW Williston Road, Gainesville, FL 32601 USA; 2 Fish and Wildlife Research Institute, Florida Fish and Wildlife Conservation Commission, 3911 Highway 2321, Panama City, FL 32409 USA; 3 Division of Habitat and Species Conservation, Florida Fish and Wildlife Conservation Commission, 3377 East U.S. Highway 90, Lake City, FL 32055 USA; 4 Department of Biology, University of Florida, 876 Newell Drive, Gainesville, FL 32611 USA

**Keywords:** Florida, Hibernation, Migration, Partial migration, *Perimyotis subflavus*, Stable isotopes, Tricolored bat

## Abstract

**Background:**

Animal migration is a widespread global adaptation by which individuals move in response to environmental conditions to reach more favorable conditions. For bats in temperate climates, migration and hibernation are often associated with each other when these bats must migrate to reach suitable overwintering sites. However, differences in movement across the geographical range of a species and the degree to which hibernation drives migratory behavior of bats in subtropical climates, where conditions may remain warm with available prey year-round, remains incomplete. Understanding the migratory strategies of subtropical bats during winter is of increasing importance as they are threatened by stressors such as disease and environmental change.

**Methods:**

We evaluated migration patterns of tricolored bats (*Perimyotis subflavus*) in Florida, USA, through analysis of stable hydrogen isotope ratios of the fur. We inferred the summer geographic origins of the fur samples and estimated the minimum distance and likely direction traveled by hibernating individuals. We used linear models to examine whether hibernation region, colony size, and an individual’s sex affected the distance traveled.

**Results:**

We sampled 111 bats hibernating at 40 sites and found that more than half (54.1%) of individuals were residents of the area in which they hibernated. We found that almost half of the sampled bats (43.2%) traveled from southern Florida to overwinter in North Florida. We also documented three individuals that traveled > 100 km from northerly origins, one of which had traveled an estimated minimum distance of 1382 km. We also found that tricolored bats moved farther to reach hibernacula in Northwest Florida and hibernacula with more populous colonies, with no difference in movement between sexes.

**Conclusions:**

Our results indicate a pattern of northward autumnal movements of tricolored bats in the subtropical southeastern portion of their range. We suggest that bats are differentially constrained at the edge of their geographical range, resulting in movement contrary to what is expected. Even though we found that few (2.7%) bats moved into Florida from farther north, those migrants can potentially transfer the fungus that causes the deadly white-nose syndrome, which does not currently occur in the state. Our results support the characterization of tricolored bats as flexible partial migrators, with a rarely exercised capacity for long-distance movements.

**Supplementary Information:**

The online version contains supplementary material available at 10.1186/s40462-022-00358-x.

## Background

Animal migration is a widespread global adaptation by which individuals move in response to environmental conditions to reach more favorable conditions, increasing personal fitness even at high energetic costs. Bat species in temperate climates often migrate seasonally to sheltered or milder environments during winter food shortages and harsh weather [1]. Migration and overwintering strategy are closely associated for many bat species outside of the tropics, with many species migrating dozens to hundreds of kilometers to reach suitable overwintering sites [1, 2]*.* Some species may be migratory in areas with high climatic seasonality and remain year-round residents in areas with milder or more consistent conditions [3, 4], but the extent to which bat species vary in migratory behavior across their geographic range is poorly understood. Differences in migratory strategies between individuals and populations may arise because of varying local weather conditions and food resources [5, 6], habitat suitability [7], and demographic and body condition factors [3, 4, 6–8], resulting in partial migration. Conversely, because transitioning between activity and extended torpor is energetically costly for hibernating bats [9], there may be fitness benefits to entering torpor even under mild environmental conditions. Caves in the southern, subtropical portion of the tricolored bat range may be unsuitable roost habitats because high temperatures prevent or disrupt torpor [10]. As transitioning between activity and extended torpor is energetically costly for hibernating bats, overwintering in unsuitably warm regions may reduce the benefits of torpor and alter movement patterns. Understanding the migratory behavior of subtropical bats during winter conditions is important as environmental change, driven by climate change and habitat loss, increasingly threatens subtropical regions [11–14].

The tricolored bat (*Perimyotis subflavus*) is a cave-hibernating species that is widespread throughout eastern North America, from Nova Scotia (Canada) to Minnesota (USA), southwest to Honduras and southeast to Florida (USA) [15–17]. Although tricolored bats were once considered common, recent stressors, including white-nose syndrome and habitat loss, have resulted in significant declines, leading to the U.S. Fish and Wildlife Service decision to propose to list the species as Federally Endangered [18]. Tricolored bats are considered obligatory hibernators, whose small size and solitary roosting habits allow them to remain in torpor for an extended time, even in warmer caves [10, 15, 16]. Historically, this species has been considered a short-distance migrant or resident species, with limited movements from summering grounds to winter roost sites (hibernacula) [15]. But recent work has demonstrated that tricolored bats may be partially migratory, with some individuals in the central and northern portion of their range migrating hundreds of kilometers north to south between summer breeding and foraging grounds and hibernacula [19, 20]. It is not well understood how environmental, climatic, and demographic variables influence the proportion of migratory and resident individuals across the range of partly migratory species.

Studies of migration by tricolored bats have focused on temperate regions of their geographic range [19, 20]. However, the southeastern edge of the species’ range is in Florida, where the climate is subtropical to humid-tropical [21]. Florida caves differ climatically and physically from caves found at more northern latitudes and higher elevations [22]. In addition, Florida has shorter, warmer winters, and insects are available most nights as prey for bats [10, 23, 24]. Yet despite such favorable winter conditions, tricolored bats exhibit the most extended torpor of any bat species in Florida [10].

Because Florida has short, mild winter conditions, it might represent a refuge for bats from white-nose syndrome (WNS), an emerging infectious disease affecting cave-hibernating bats species. The subtropical conditions may reduce the impacts of WNS since lower fat stores are required to survive the short winter and foraging is possible on most nights. White-nose syndrome results from infection by the fungus *Pseudogymnoascus destructans* (*Pd*) and affects the hibernation and hydration cycles of hibernating bats, causing them to arouse more often, depleting crucial fat reserves and ultimately resulting in death [25–28]. In areas where WNS has been documented to the north, tricolored bat populations have declined by > 90% [29]. Although spread models indicated that *Pd* would reach Florida as early as 2016 [30], it has yet to be detected on a bat or at a hibernaculum in the state (Smith, unpublished data). Where WNS occurs*, Pd* spores are present in the hibernaculum substrate in summer [31] and could be transferred between bats. Natural movements of infected bats at stopover sites, winter swarming caves, and hibernacula are hypothesized to be the main natural means of *Pd* transmission [32–34]. Because hibernacula used by bats from multiple breeding and foraging grounds are more susceptible to *Pd* [31, 35], it is becoming increasingly salient to determine the degree to which migration might contribute to the spread of *Pd* to Florida.

The primary objective of this study was to determine the probable summer origins of tricolored bats that use hibernacula at the southeastern extent of their range. To do this, we measured the stable hydrogen isotope ratios (*δ*^2^H) within fur of tricolored bats at winter hibernacula. Because animal tissue reflects the stable isotope ratios of the local food web at the time and location at its formation, measurements of stable isotope ratios of animal tissue can be used to infer an animal’s geographic origin [20, 36]. A secondary objective was to evaluate the potential for tricolored bats to spread *Pd* to Florida hibernacula by long-distance autumn migration through *Pd*-positive regions. We hypothesized that bats hibernating in Florida would include a mix of non-migratory residents and bats that migrate to Florida from sites farther north, which are potential vectors of *Pd* for the state. Finally, we evaluated the degree to which migratory behavior may have been affected by hibernaculum region, colony size (number of bats detected at a hibernaculum), or an individual’s sex.

## Methods

### Sample collection

We collected samples and data during two time periods in the annual cycle of the tricolored bat: winter (1 January–15 March 2018), when tricolored bats have completed any autumn migration from their summering grounds, and the time of the summer molt, when new fur has recently been grown, and presumably reflects the stable hydrogen isotope values of the geographic region. We relied on an estimate of molt period from Fraser et al. [20], 23 June–16 October 2017, which was calculated based on the interval within the annual cycle in which the stable hydrogen isotope measurements of tricolored bat fur most closely matches that of precipitation at the sampling location. Collecting samples in the winter months allows for inference of the autumn migratory movements between the inferred summer molt location and hibernaculum. Samples collected during the molt were considered known-origin and were used to validate model performance and quantify the relationship between *δ*^2^H values of local precipitation (*δ*^2^H_precip_) and those of bat fur (*δ*^2^H_fur_).

**Fig. 1:**
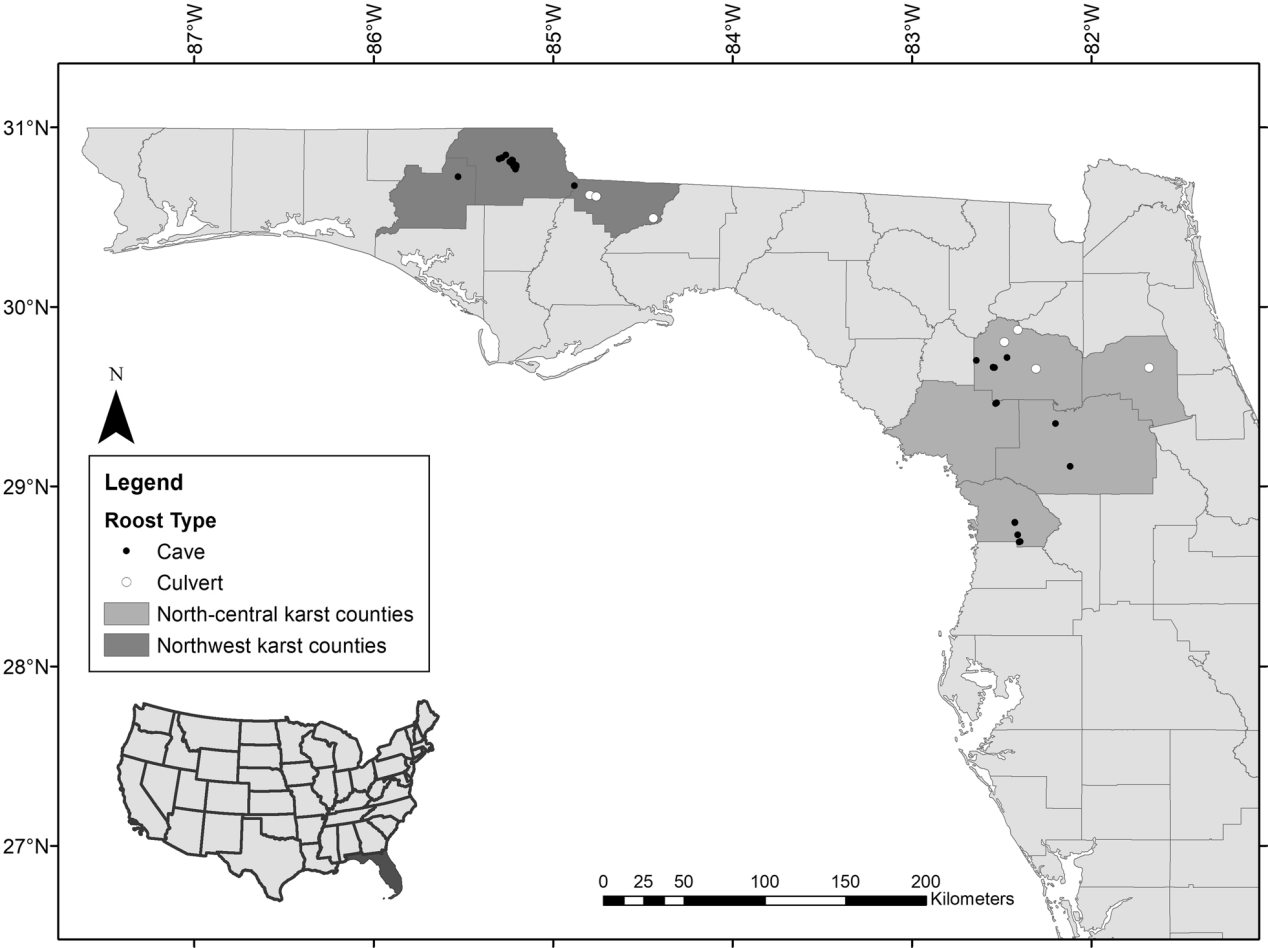
Locations of tricolored bat (*Perimyotis subflavus*) hibernacula included in this study. Cave locations (*n* = 33) are indicated with black dots, culvert locations (n = 7) with white. Florida counties containing two karst regions are highlighted in lighter and darker shades of gray for North-central and Northwest regions, respectively

In winter 2018, we surveyed 33 cave and 7 culvert (concrete box or pipe structures > 1 m tall) hibernacula in the karst regions of Northwest (Gadsen, Jackson, and Washington counties) and North-central (Alachua, Citrus, Levy, Marion, and Putnam counties) Florida as part of the state’s ongoing bat-monitoring efforts (Fig. 1). In both regions, most hibernacula were in upland hardwood, mixed wetland hardwood, and mixed hardwood–coniferous forest habitats [37]. In Northwest Florida, the mean high temperature in winter is 18.6 °C; temperatures are below freezing 29 days a year, on average (Tallahassee weather station) [38]. In North-central Florida, the mean high temperature in winter is 20.9 °C; temperatures are below freezing 16 days a year, on average (Gainesville weather station) [38].

During winter surveys, we systematically searched for bats in all accessible portions of each cave and culvert and counted each tricolored bat observed. We grouped sites by colony size as large (≥ 20 tricolored bats) or small (< 20 tricolored bats) based on standards defined by the Florida Fish and Wildlife Conservation Commission and by karst region as Northwest or North-central (Fig. 1). We reduced disturbance to the bats during surveys by using red lights, minimizing noise, and limiting the time spent inside each hibernaculum. We minimized the potential risk of transferring *Pd* by following accepted decontamination protocols [39]*.* We sampled fur from 1 to 16 bats at each site, and to limit disturbance, we sampled no more than half the bats in the small colonies and as many bats as possible in the large colonies without remaining in one area of the cave > 30 min. For each tricolored bat sampled, we cut approximately 1 mg (a 1 cm strip) of fur from the area between the scapulae and recorded location, sex, and age. Each fur sample was placed in a paper envelope and stored in a cool, dry place. All bats sampled in winter were assumed to be adults as it is no longer possible to definitively classify age [40].

To augment existing records of summer *δ*^2^H values of tricolored bat fur, we requested samples from ongoing monitoring efforts throughout the species’ geographic range. We received fur samples from tricolored bats captured in mist nets collected during the molt period in the 2015 and 2017 monitoring seasons. Samples were obtained from northwestern Arkansas, northern West Virginia, northern Maryland, Central Kentucky, and Central Florida.

The same standardized protocol used for winter sampling was provided to all volunteers providing fur from mist-netted bats in summer. We visually confirmed each sample as tricolored bat fur based on the distinctive three-colored banding pattern that is unique to the species. Each fur sample was associated with additional metadata including sampling location and bat sex and age class (juvenile vs. adult).

### Stable hydrogen isotope analysis

Stable hydrogen isotope analyses were conducted at the Central Appalachians Stable Isotope Facility (CASIF) at the University of Maryland Center for Environmental Science Appalachian Laboratory (Frostburg, MD, USA) following the sample preparation and *δ*^2^H measurement protocols detailed in [41]. Fur samples were cleaned using 1:200 Triton X-100 detergent, rinsed with 100% ethanol, and allowed to dry at ambient air temperature [42]. Samples were analyzed alongside international standards (USGS42; USGS43; CBS [Caribou Hoof Standard]; KHS [Kudu Horn Standard]; [42]) and an internal keratin standard (porcine hair and skin, product #K3030; Spectrum Chemicals, New Brunswick, NJ, USA) for a comparative equilibration [43]. Samples and standards were exposed to ambient air for > 72 h before analysis to allow equilibration of exchangeable hydrogen in the keratin standard. Samples were analyzed using a ThermoFisher high-temperature-conversion/elemental-analyzer pyrolysis unit interfaced with a ThermoFisher Delta V + isotope-ratio mass spectrometer. Resulting *δ*^2^H values were normalized to the Vienna Standard Mean Ocean Water–Standard Light Antarctic Precipitation (VSMOW-SLAP) scale using USGS42, USGS43, CBS, and KHS (*δ*^2^H values of nonexchangeable hydrogen of these standards are − 72.9, − 44.4, − 157.0, and − 35.5‰, respectively) [43, 44]. Analytical precision of the internal keratin standard was 2.3‰ for *δ*^2^H.

To construct origin models for bat fur collected in the winter, we relied on a data set of known-origin samples representing *δ*^2^H_fur_ samples obtained during the interval of fur growth (summer molt [20, 38]). We supplemented our known-origin *δ*^2^H_fur_ measurements with data from [45] to increase sample size and geographic coverage (Additional file 1: Figure S1). To integrate the data sets of *δ*^2^H_fur_ values of tricolored bat fur from [45], which were obtained using standards different from those used at CASIF, we used a calibration chain transformation to adjust the scale reflecting in-house standards (cow hoof and bowhead whale baleen standards [45]) to one reflecting international standards (VSMOW-SLAP) [46], relying on the functions and data sets available in the assignR R package (v. 1.2.1.9001) [47].

Next, we evaluated several candidate spatial models of precipitation *δ*^2^H values (*δ*^2^H_precip_) for relative power to predict the *δ*^2^H values of tricolored bat fur sampled during molt (*δ*^2^H_fur_). We tested *δ*^2^H_precip_ spatial models (isoscapes) reflecting annual, growing-season, and multimonth summer temporal extents including assignR’s global growing season H isoscape [47] and IsoMAP jobs 70,052, 70,055, 70,064, 73,715, 66,098, and 66,100 [48]. Each isoscape was reprojected to a North America Albers equal-area conic projection, then cropped to the extent of continental North America land mass [49]. Because we transformed the known-origin data set from [45] to a different standard scale, and because we augmented the data set with additional individuals sampled during the period of fur growth, we fit new regressions relating *δ*^2^H_precip_ at the sampling site to known-origin *δ*^2^H_fur_. Variation in the relationship between *δ*^2^H_precip_ and *δ*^2^H_fur_ may exist across age [50] and sex [20, 41, 51] due to differences in molt timing and isotopic discrimination. We try to account for such variation by using a broad dataset of individuals sampled during their molt to model this relationship, and then incorporating variation in that relationship into subsequent steps of assignment mapping [52]. We did so using a bootstrapped standard major-axis regression as in [52] using the smatr R package [53], which offers the advantage of being a two-way regression, permitting a two-way translation between *δ*^2^H_fur_ and *δ*^2^H_precip_. We used a bootstrapped resampling approach to regression fitting, in which data points were iteratively resampled with respect to sampling location for more than 5000 replications, returning estimated model coefficients. We fit models to each isoscape estimate of *δ*^2^H_precip_, selected the regression with the highest $\overline{{R^{2} }}$, and applied the estimated model parameters to transfer the *δ*^2^H_fur_ (VSMOW) values for the sampled individual to the expected *δ*^2^H_precip_ (VSMOW). To confirm that analysis laboratory was not driving any variation in transfer function fit, we applied a t-test of transfer function model residuals with respect to analysis laboratory for samples collected during the molt period.

We created individual probability-of-origin maps using the R package isocat’s modeling capabilities. To do so, we incorporated individual-level expected *δ*^2^H_precip_ (VSMOW), a general measurement-error metric of the $\overline{sd}$ of the *δ*^2^H_fur_ to *δ*^2^H_precip_ regression, and the top-performing isoscape and its associated error surface. We incorporated Bayesian priors to reflect binary values indicating areas within and outside of the species range (probabilities of 1 and 0, respectively). We generated the priors’ surface by buffering the IUCN range map [54] by 10 km and masking to the extent of temperate terrestrial North America.

We estimated the minimum distance traveled by each individual through a nonparametric bootstrapping method that incorporates model performance on a test set of known-origin individuals, as proposed by [52]. In brief, we estimated the odds-ratio (OR) of each probability-of-origin value *i* of each probability surface *k* as $OR = {{\frac{i}{1 - i}} \mathord{\left/ {\vphantom {{\frac{i}{1 - i}} {\frac{\max \;(k)}{{1 - \max \;(k)}}}}} \right. \kern-\nulldelimiterspace} {\frac{\max \;(k)}{{1 - \max \;(k)}}}}$ [36]. We then determined the odds of origin of each known-origin individual at its sampling location. We resampled the OR_known-origin_ values 100,000 times with replacement and calculated the proportion of the number of times each OR value from the surface was greater than the resampled value. We then reported the proportion of times each OR value was equal to or greater than the corresponding simulated OR_known-origin_ value, resulting in an OR-simulation surface with probabilities of origin ranging from 0 to 1. We estimated minimum distance traveled for each OR-simulation surface at a threshold of 0.25 (i.e., at a 75% likelihood that an OR value would fall within the distribution of the OR_known-origin_ value) and calculated the minimum distance between that point and the sampling site on an ellipsoid (a geodesic) using the geosphere R package [55]. We binned individuals by minimum distance traveled as follows: residents, < 100 km; regional migrants, 100–1000 km; and long-distance migrants, > 1000 km [7]. We assumed that any movements between summer and winter represent seasonal migration, with the possible factor of juvenile dispersal incorporated as part of broad seasonal shifts in the population of tricolored bats.

Next, we estimated the general directionality of origin for regional and long-distance migrants. To do so, we calculated the bearing of each origin (angle θ values ranging from − 180 to 180°) relative to the site at which an individual was sampled, rounded to the nearest whole θ. The θ value containing the greatest mean probability of origin was considered the most likely bearing ($$\overset{\lower0.5em\hbox{$\smash{\scriptscriptstyle\frown}$}}{\theta }$$) of origin. We also binned individuals into two direction-linked groups: those with northerly origins for individuals with $$\left| {\overset{\lower0.5em\hbox{$\smash{\scriptscriptstyle\frown}$}}{\theta } } \right| < 90$$; and southerly origins, where $$\left| {\overset{\lower0.5em\hbox{$\smash{\scriptscriptstyle\frown}$}}{\theta } } \right| \ge 90$$. We report direction of origin for individuals with strong evidence of movement from a distant summer origin, for which minimum distance traveled was ≥ 100 km.

We tested for the effects of hibernaculum characteristics (karst region and bat colony size) and sex on the minimum distance traveled from summering grounds to hibernaculum using both *t* tests and a linear modeling framework. First, we individually tested the differences in minimum distance traveled across hibernacula characteristics and sex using Yuen’s test for trimmed means [56], which is robust for unequal population variances and under long-tailed distributions [57]. To test the predictive and explanatory power of variables with respect to distance migrated, we fit generalized linear models. We considered hibernaculum region, colony size, and sex as predictors and minimum distance traveled as a response variable. We applied the dredge function from the R package MuMIn [58] to examine all possible models and used backward model selection to select the top-performing model. Models were ranked using Akaike’s information criterion [59]; we discarded competing models if variance-inflation factors were ≥ 5 [60].

All statistical analyses were conducted in R version 4.0.2 (2020-06-22). We relied heavily on the R packages isocat [52, 61], raster [62], tidyverse [63], assignR [49], and ggstatsplot [64].

## Results

**Fig. 2:**
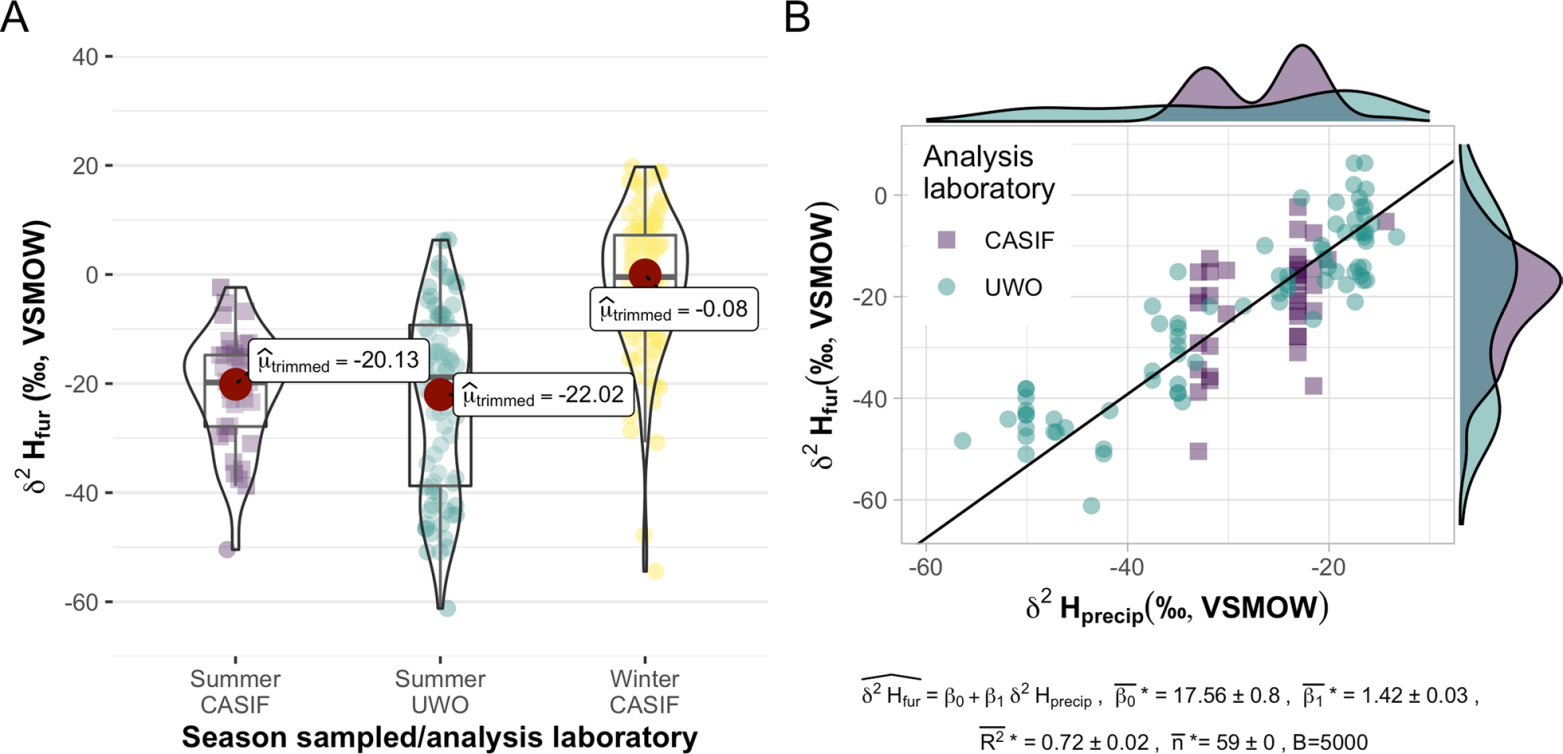
A summary of δ^2^H_fur_ values with respect to season of sampling, stable isotope laboratory, and of δ^2^H_precip_ at site of sampling. Panel **A** shows the δ^2^H_fur_ (‰, VSMOW-SLAP) values for known-origin summer bat fur analyzed at the University of Western Ontario (UWO; *n* = 78) and Central Appalachian Stable Isotope Facility (CASIF; *n* = 37) respectively, alongside unknown-origin winter bat fur analyzed at CASIF (*n* = 111). Panel **B** shows the inferred relationship between δ^2^H_precip_ (top-performing isoscape was a June–August δ^2^H_precip_ model, IsoMAP job 66,098) and known-origin summer δ^2^H_fur_ (‰, VSMOW-SLAP). The results of a bootstrapped linear regression, with subsampling iterated with respect to sampling site, are reported below, with mean parameter estimates plotted on the scatter plot

We obtained and analyzed fur samples from 37 tricolored bats collected during the molt period, which ends prior to the expected period of autumn migration. Samples from molt period bats had *δ*^2^H_fur_ ranging from − 50.40 to − 5.21‰. After synthesis with the chain-link-transformed data set from [45] (*n* = 78), we enlarged the known-origin bat fur data set to incorporate data from 115 individuals (*n*_female_ = 49; *n*_male_ = 66), representing 53 sites across 19 states (*n*_Florida_ = 4) throughout the eastern range of the tricolored bat. We detected no difference between summer δ^2^H_fur_ (VSMOW-SLAP) values (*p*_Holm-corrected_ > 0.05, Fig. 2a) or model residuals (*p*_Welch_ > 0.05, Additional file 1: Figure S2) across analysis laboratories (*p*_Holm-corrected_ > 0.05; Fig. 2a), indicating successful transformation to reflect the same isotopic standard scales.

We analyzed 111 fur samples collected in winter (*n*_female_ = 68; *n*_male_ = 33, *n*_unknown_ = 10; *n* individuals/site range, 1–16, μ = 2.6) from 40 hibernacula (*n*_cave_ = 33; *n*_culvert_ = 7) in the karst regions of Northwest and North-central Florida (Fig. 1). Sex was not recorded for 10 bats found dead soon after a flood because their condition had deteriorated. We sampled 38 individuals from 19 sites in North-central Florida and 73 individuals from 21 sites in Northwest Florida. We sampled 104 individuals from cave sites and 7 individuals from culvert sites. Values of δ^2^H_fur_ ranged from − 54.42 to 19.72‰ (Fig. 2a).

To determine which transfer functions best related known-origin *δ*^2^H_fur_ samples to modeled δ^2^H_precip_, we fit candidate-transfer functions, bootstrapped with resampling, to six candidate isoscapes. The top-performing isoscape was a June–July–August precipitation model (IsoMAP job 66,098). Bootstrapped parameter estimates were $\overline{{\beta_{0} }} * = 17.56 \pm 0.8\;{\text{and}}\;\overline{{\beta_{1} }} * = 1.42 \pm 0.03$, where $\widehat{{\delta^{2} {\text{H}}_{{{\text{fur}}}} }} = \beta_{0} + \beta_{1} \delta^{2} {\text{H}}_{{{\text{precip}}}}$ $\left( {B = 5000,\;\overline{{{\text{R}}^{2} }} * = 0.72 \pm 0.02,\;{\overline{\text{n}}}\;{* = 59} \pm {0}} \right)$, *δ*^2^H_fur_ sample values were translated to estimated *δ*^2^H_precip_ values using mean parameter estimates (Fig. 2b).

**Fig. 3:**
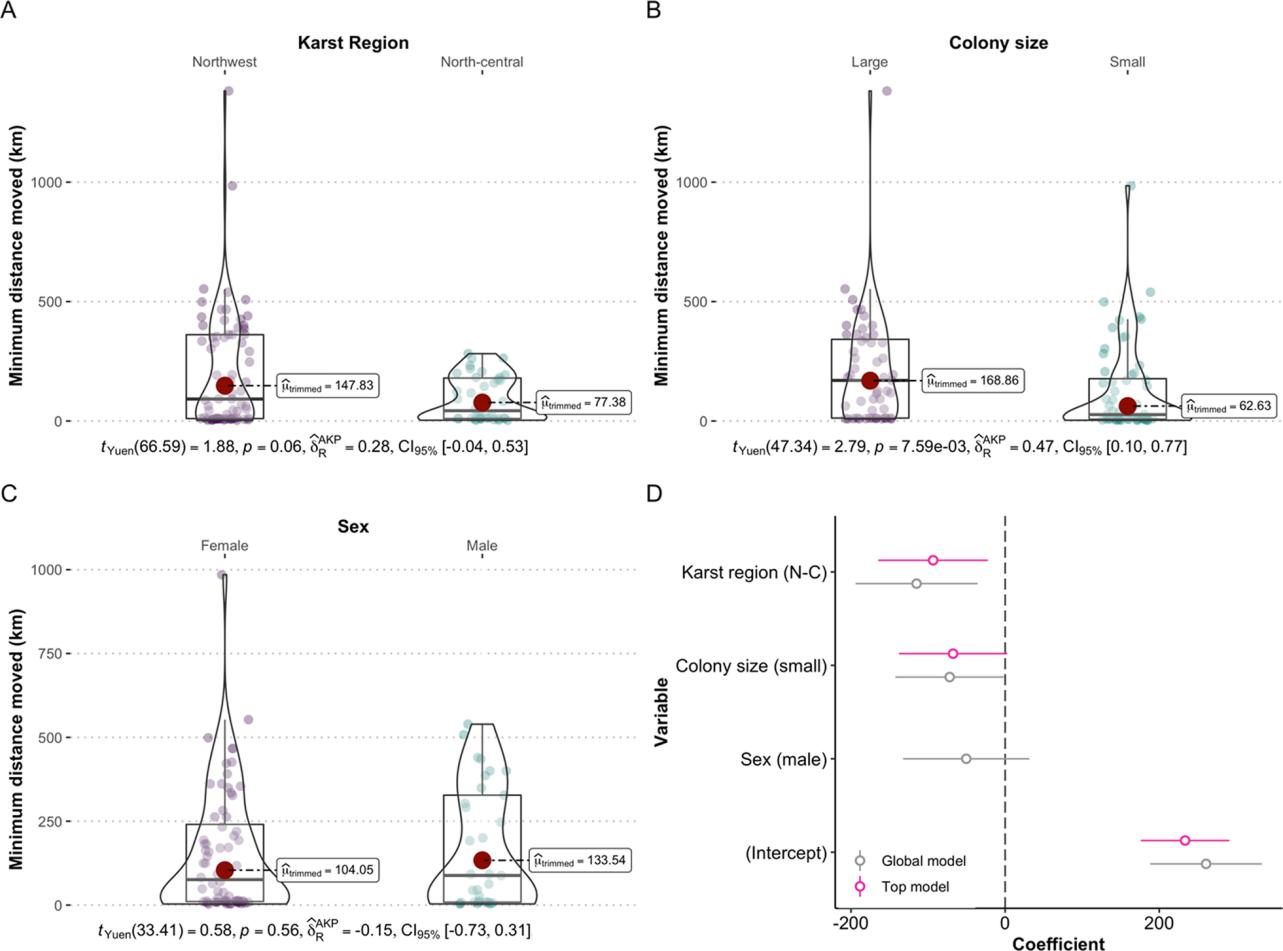
Results of Yuen’s trimmed-mean *t*-tests testing minimum distance moved (km) with respect to **A** karst region, **B** colony size, and **C** sex for tricolored bats (*Perimyotis subflavus*) in Florida. Panel **D** shows model coefficients for global (gray) and top-performing (pink) generalized linear models

**Fig. 4:**
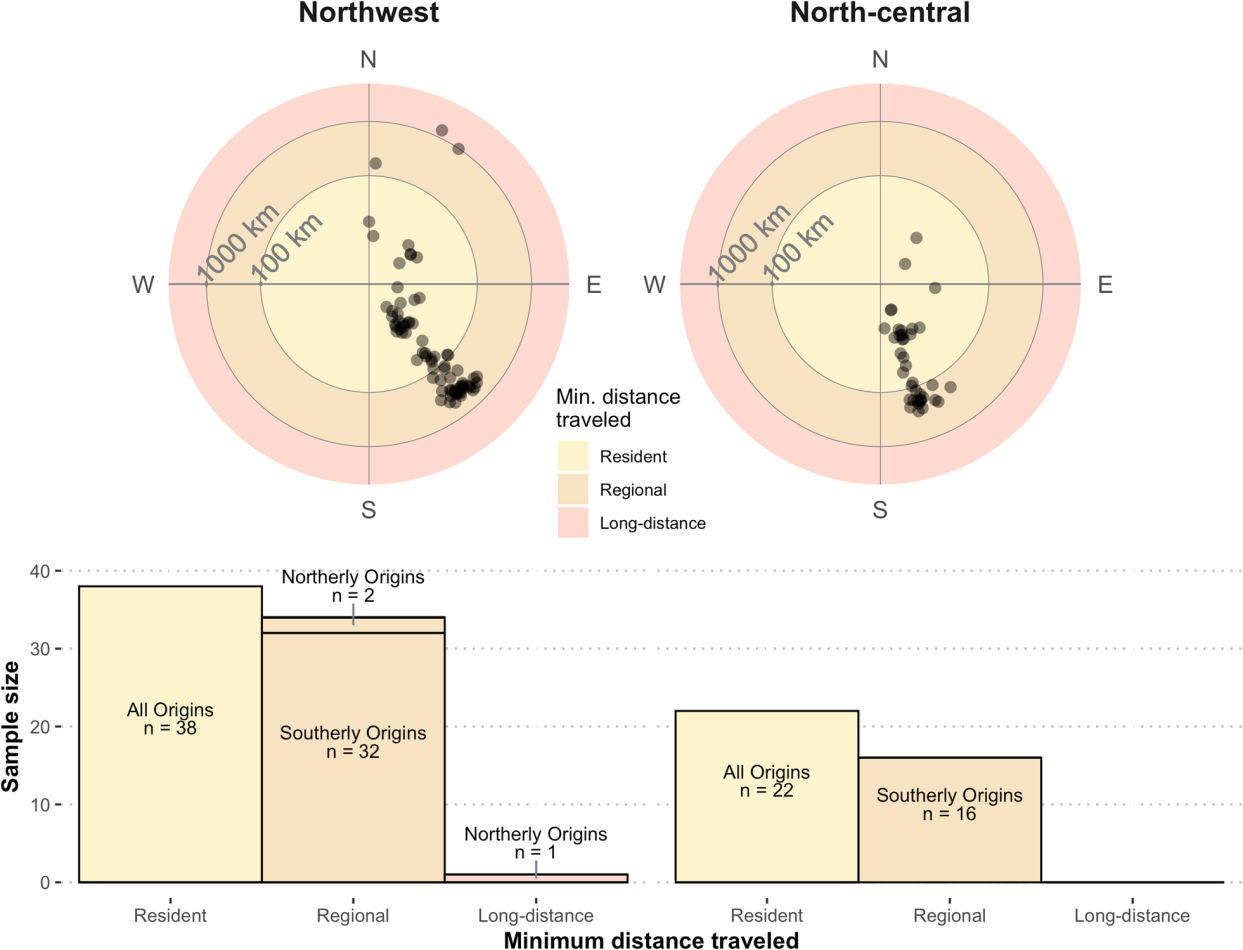
At top, the estimated angle of origin for each hibernating tricolored bat (*Perimyotis subflavus)* sampled in Northwest and North-central Florida in winter 2018. The most likely direction $$\left( {\overset{\lower0.5em\hbox{$\smash{\scriptscriptstyle\frown}$}}{\theta } } \right)$$ of the summering location where fur was grown is indicated with respect to winter location. Distance of the point from the center indicates minimum distance traveled from summering grounds to the hibernaculum, with highlighted bins illustrating resident bats (minimum distance traveled < 100 km), regional migrants (minimum distance traveled 100–1000 km), and long-distance migrants (minimum distance traveled > 1000 km). At bottom, we summarize the general direction of origin for each group of bats, reporting northerly or southerly origins when minimum evidence of movement is > 100 km

**Fig. 5:**
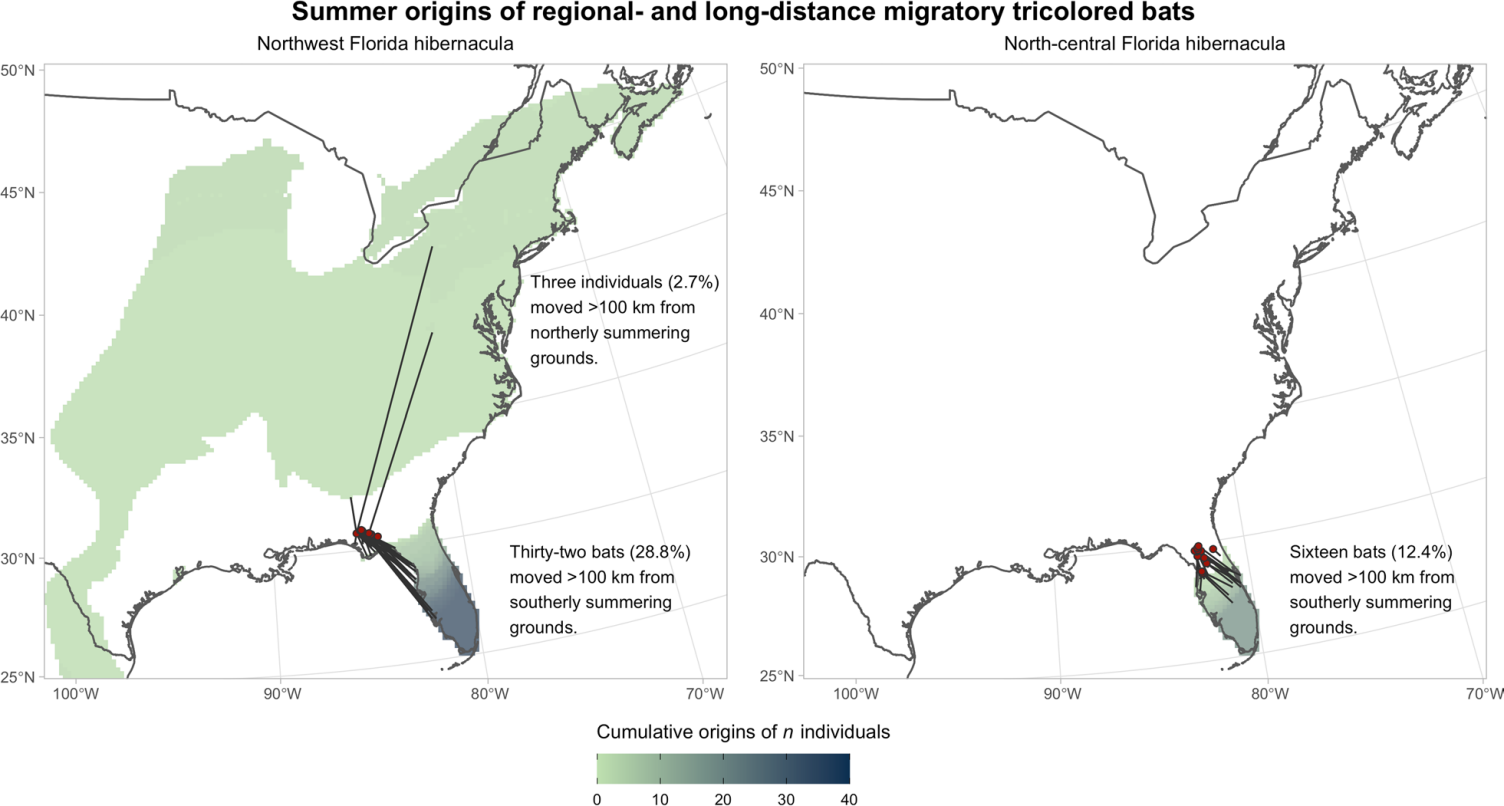
Aggregated summer origins of 51 regional and long-distance-migrating tricolored bats (*Perimyotis subflavus*) hibernating in Florida caves and culverts. For each individual origin, we thresholded the odds-ratio simulation surfaces at 0.25, for which 75% of known-origin individuals would be expected to be included in the area above that threshold. We aggregated these surfaces to find the cumulative number of origins expected at a given location, with respect to hibernacula region: the origins of bats hibernating in Northwest Florida hibernacula (left) or North-central (right). Lines indicate the cell of the nearest likely summer origin of a referenced individual. Hibernaculum locations are indicated with red dots

Odds-ratio probability-of-origin surfaces were generated for all individuals. As expected, odds of origin at the sampling site for known-origin individuals were high: μ_OR_ = 0.73, σ_OR_ = 0.22, median_OR_ = 0.82, range 0.21–0.9995. After OR-simulation adjustment to account for origin model error, the median minimum distance from summering grounds to sample site at the OR-simulation threshold of 0.25 was 9 km (μ = 84.6 km, σ = 201 km) for known-origin individuals and 82 km (μ = 164 km, σ = 213 km) for winter-sampled individuals. Of the bats that wintered in Florida, more than half (*n* = 60; 54%) were residents with a minimum distance traveled of < 100 km; 50 (45%) were regional migrants (minimum distances traveled 100–1000 km), and 1 individual was a long-distance migrant (> 1000 km; Fig. 3). Most regional migrant bats moved north in autumn (Figs. 4 and 5); with 48 (96%) individuals with fur samples grown south of their winter sampling location. Three individuals hibernating in the northwest karst region had grown their fur farther north, outside of Florida (Fig. 5): two regional migrants with minimum distance traveled estimates of 170 km and 985 km (sex female and unknown, respectively) and one long-distance migrant that traveled an estimated minimum of 1382 km (sex unknown).

We tested for the effect of karst region, colony size, and sex on individual minimum distance traveled and found the top-performing model was structured as: DistanceTraveled ~ ColonySize + KarstRegion (Fig. 3d; R^2^ = 0.107, estimated intercept = 233.53). Karst region was an important predictor of distance traveled. Individuals that wintered in North-central Florida moved approximately 93 km (standard error = 36.33, *p* = 0.012) less than individuals that wintered in Northwest Florida. We interpret the lack of significance using a trimmed-means test as suggesting that this difference is driven by long-distance migrants at the tail of the distribution (*t*_Yuen_ = 1.88, *p* = 0.065; Fig. 3a). Colony size had a marginal effect on the distance moved by individuals; bats at more populous hibernacula had moved slightly farther (size: small parameter estimate =  − 67.53, standard error = 35.84, *t*-value =  − 1.88, *p* = 0.06; trimmed-means comparison *t*_Yuen_ = 2.79, *p* < 0.01; Fig. 3b). Sex was not a meaningful predictor of distance traveled (not included in top model, *t*_Yuen_ = 0.58, *p* > 0.1; Fig. 3c).

## Discussion

We found a pattern of northward autumnal movements by many tricolored bats in the subtropical southeastern portion of their range. Although, based on isotopic evidence, slightly more than half (54.1%) of the bats that we sampled during winter in Florida hibernacula did not travel beyond 100 km, almost half (43.2%) of the bats summered > 100 km south of their hibernation sites. In contrast, in the northeastern and north-central United States, regional southward autumnal migration was documented in almost a third (27.6%) of tricolored bats, with very few moving northward [20], demonstrating that individuals were more likely to migrate equatorward in autumn if they had summered at higher latitudes near the edge of their range. Those southward movements were hypothesized to increase overwinter survivorship of tricolored bats because hibernating in more southerly regions would result in a shorter hibernation period that requires less fat reserves for successful hibernation [9, 20, 65]. We suggest that tricolored bats may be constrained by winter conditions at both the northern and southern edges of their geographic range, encouraging partial seasonal migration of bats inward from both edges of their range.

Northward migration in the northern hemisphere in autumn is uncommon in most animals. Rare examples of northward migration have been documented in bird species, usually when such migration enables a species to exploit food resources. For example, long-legged buzzards (*Buteo rufinus*) migrate northward as the leaves fall off trees, improving foraging success [66], and thin-billed prions (*Pachyptila belcheri*) migrate northward to productive feeding areas in the ocean [67]. Unlike these birds, tricolored bats near the southeastern edge of their range are departing a region with a shorter winter and more abundant and consistent winter foraging resources [68, 69]. Since roost temperatures above a certain threshold increases the energetic cost of torpor, causing bats to deplete their fat stores too quickly [70], it is more likely that bats migrate northward to locate suitable winter roosts. This necessity has been reported in gray bats (*Myotis grisescens*), which have specific microclimatic requirements for maternity and winter seasons. It has been hypothesized that caves in the extreme southern range of the gray bat are too warm in winter for gray bats to sustain torpor, resulting in movements > 400 km northward in autumn [71]. Additionally, Schreiber’s bats (*Miniopterus schreibersii*) make short northward migrations in the northern hemisphere to roost in cooler hibernacula instead of selecting for preferred foraging habitats [72]. Although tricolored bats are more tolerant of warmer hibernacula than many other species [10], they are more abundant in cooler caves in subtropical regions [73] and may also benefit from northerly movements to ideal microclimates. Other bat species are known to migrate north as part of a radial migration, or movement from all directions, to reach the winter roost [74]. However, we do not believe that the northward movements we documented is part of a radial migration because we would expect to see equal numbers of bats moving from north to south.

Although South Florida offers nearly year-round foraging resources for bats that may allow them to remain active during winter, it may be biologically advantageous for bats to enter torpor during their annual cycle, which requires that they locate sites with suitably cooler temperatures that allow them to lower their body temperature [10]. Female tricolored bats mate in the fall prior to hibernation and store viable sperm in their oviducts until spring, when they ovulate and the eggs are fertilized [1, 75, 76]. Because spermatozoa may disappear quickly from the reproductive tracts of active individuals with a higher body temperature [77], torpor may be necessary for successful reproduction in this speices. Since many roosts in South Florida are likely too warm to support torpor, female bats may need to move north to cooler sites. But we did not find differences in movement between male and female tricolored bats, suggesting that the driver of northward autumn migration may not be sex-linked. Since tricolored bat mating occurs in the autumn at congregations at hibernacula, or swarming sites, both male and female bats may migrate toward autumn swarming locations in north Florida and beyond. Northward movement from South Florida of females and males is supported by acoustic monitoring in that detected tricolored bats only during the summer wet–warm season [78]. Another explanation might be sensitivity to weather fluctuations. In South Florida, even rare instances of inclement weather (e.g., infrequent cold snaps and low abundance of winter prey) might make roosting in less sheltered sites that occur there riskier. Because caves in South Florida are uncommon, frequently disturbed, with an unstable microclimate [79], severe weather may prompt tricolored bats to migrate north to more suitable hibernacula. Broadly, we suggest that some tricolored bats migrate northward in autumn to reach cooler, more stable roosts in northern Florida, where conditions are apparently more suitable for torpor.

Bats that moved more than 100 km from South Florida breeding and foraging grounds preferentially selected hibernacula in Northwest Florida, where twice as many bats overwintered as in North-central Florida (Fig. 5). As the northwest hibernacula are approximately 330 km farther from South Florida than are north-central hibernacula (Fig. 1), most regional migrants seem to be preferentially selecting more distant hibernacula over closer ones. Microclimate is believed to have a strong impact on hibernaculum selection because it helps determine energy savings and water loss and, therefore, winter survival [80]. Hibernaculum temperature varies with latitude [23], and hibernacula in Northwest Florida are on average 3.5 °C cooler than those in North-central Florida (Smith, unpublished data). Tricolored bats in Florida increase in abundance in winter at caves with a cooler temperature [73], and bats may be moving farther to sites in Northwest Florida, or sites farther north outside of the state, where there are more hibernacula with the preferred cooler microclimate.

We found that tricolored bats in more populous winter colonies had moved farther from their summer origins, while bats in smaller winter colonies were more likely to be residents. Hibernacula with ideal conditions (i.e., stable, cool temperatures, high humidity, limited disturbance) likely attract more bats for hibernation because they result in a better body condition at the completion of winter. Temperatures at more populous hibernacula were 2.7 °C cooler than those at smaller hibernacula, promoting deeper torpor (Smith, unpublished data). Therefore, some bats may have adapted to expend more energy to reach a hibernaculum if energy savings in the long term offset the energy costs of reaching the site. Additionally, hibernating in more populous colonies provides benefits including reduced predation risk, lower thermoregulation costs, and increased social benefits [81].

We found that bats moved different distances depending on karst region or colony size and this may have implications for genetic diversity, disease transmission, and parasite spread. As individuals move through swarming sites to hibernacula, contact between individuals is increased and may facilitate the transmission of diseases and parasites among populations [82, 83]. This could affect hibernacula where individuals from various summer origins come together more strongly. Additionally, individuals moving long distances to reach a hibernaculum may increase genetic diversity since swarming and mating occur on the wintering grounds, and individuals originating from different summering grounds may increase gene flow across populations [2, 84]. Increasing genetic diversity in a population can help stabilize demographics and, especially, reduce mortality from diseases, such as white-nose syndrome if it reaches the state [32].

*Pd,* a cold loving fungus found in caves, has been spreading throughout North America at a rate expected of the short- to middistance migratory movements of cave-hibernating bats [30, 85]. Although WNS is present across most of eastern North America and as far south as Texas, it has not been detected in Florida, despite close proximity (~ 250 km) to *Pd*-positive sites in Georgia, Alabama, and Mississippi. Even though we found that only a small proportion of sampled tricolored bats in our study moved from likely *Pd-*positive regions north of Florida, either as a migratory movement or juvenile dispersal (Fig. 5a), the possibility that those bats were exposed to *Pd*-positive substrates and bats during migration and swarming is significant [31]. Because caves in Florida have a microclimate suitable for *Pd* fungal growth [73], it is unclear why the fungus has not been detected in the state. We suspect the limited migratory connectivity we found between Florida and northern *Pd*- positive regions may be inhibiting the southerly spread of *Pd*. But, because some tricolored bats from northern regions do move into Florida, monitoring and surveillance efforts for *Pd* should continue, especially in the more populous northwest caves used by bats from multiple migratory pathways. Additionally, the frequent northward movements we observed demonstrates the importance of understanding movement patterns across the geographical range of a species to better inform models of disease spread and evaluate risk to different regions.

Stable hydrogen isotope analysis revealed that tricolored bats hibernating at their southeastern range edge engage in various migratory strategies. We found that partial migration was a common strategy of tricolored bats; many hibernacula contained residents as well as northward and southward migratory individuals. We found that almost half of the sampled tricolored bats moved northward, contrary to previous studies [19, 20]. This demonstrates the variation in movement across the geographic range of the species and the need for increased study along the range edge of a species. Although fewer than half of the individuals moved a detectable distance between summer and winter grounds, we also found evidence of regional and long-distance migratory movements. The longest movement (> 1300 km) we estimated for a tricolored bat is longer than had been documented for this species [19, 20]. Tricolored bats had been thought to be predominantly nonmigratory [15], but our findings support recent isotopic and telemetry evidence of regional migratory movements [19, 20]. We affirm the characterization of these small bats as flexible partial migrators, even in the subtropical environment of Florida with suitable year-round foraging conditions. It is likely that the selective advantage of moving to suitable roost sites and increasing energetic savings during torpor influences migratory behavior in a subset of the population.

## Supplementary Information

**Additional file 1**. **Figure S1**: A map of sampling locations of known-origin (sampled during summer molt period) tricolored bat fur used as a model-fitting and testing set in this analysis. The IUCN range of the tricolored bat is highlighted, and the colored background highlights geographic variation in precipitation stable hydrogen isotope values. Point shape and color show the analysis laboratory at which samples were analyzed. **Figure S2**: Residuals from the selected best-fit transfer function, plotted with respect to season of sampling and analysis laboratory. Significant three-way interactions are highlighted (pHolm-corrected ≤ 0.05)

## Data Availability

Code used to generate the figures and analysis is available at https://github.com/cjcampbell/PESU_migration_ms, and data and code are available on Zenodo at https://doi.org/10.5281/zenodo.7067284.

## Abbreviations

WNS: White-nose syndrome
*Pd*: *Pseudogymnoascus destructans*
CASIF: Central Appalachians Stable Isotope Facility
VSMOW-SLAP: Vienna Standard Mean Ocean Water–Standard Light Antarctic Precipitation
OR: Odds-ratio
