# Supplementary material for: Tricolored bats at a southern range edge exhibit partial migration northward in autumn
Source: Mov Ecol. 2022 Dec 2;10:56. doi: 10.1186/s40462-022-00358-x (PMC9717247; doi:10.1186/s40462-022-00358-x)
Supplement: Supplementary file 1 — Additional file 1. Figure S1: A map of sampling locations of known-origin (sampled during summer molt period) tricolored bat fur used as a model-fitting and testing set in this analysis. The IUCN range of the tricolored bat is highlighted, and the colored background highlights geographic variation in precipitation stable hydrogen isotope values. Point shape and color show the analysis laboratory at which samples were analyzed. Figure S2: Residuals from the selected best-fit transfer function, plotted with respect to season of sampling and analysis laboratory. Significant three-way interactions are highlighted (pHolm-corrected ≤ 0.05) [file 40462_2022_358_MOESM1_ESM.docx]

**Supplemental Information 1**


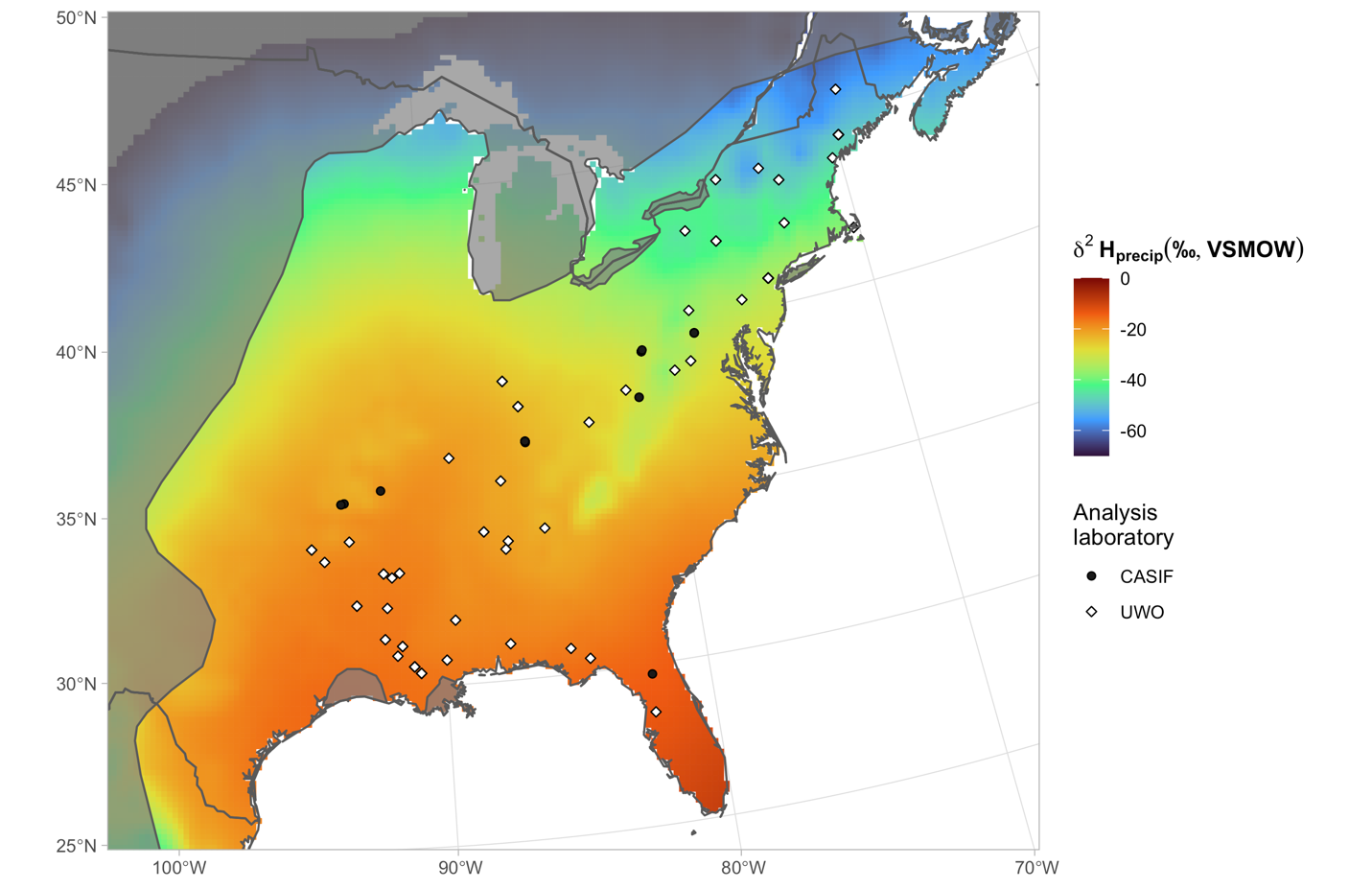
Supplemental Figure 1. A map of sampling locations of known-origin (sampled during summer molt period) tricolored bat fur used as a model-fitting and testing set in this analysis. The IUCN range of the tricolored bat is highlighted, and the colored background highlights geographic variation in precipitation stable hydrogen isotope values. Point shape and color show the analysis laboratory at which samples were analyzed.

**
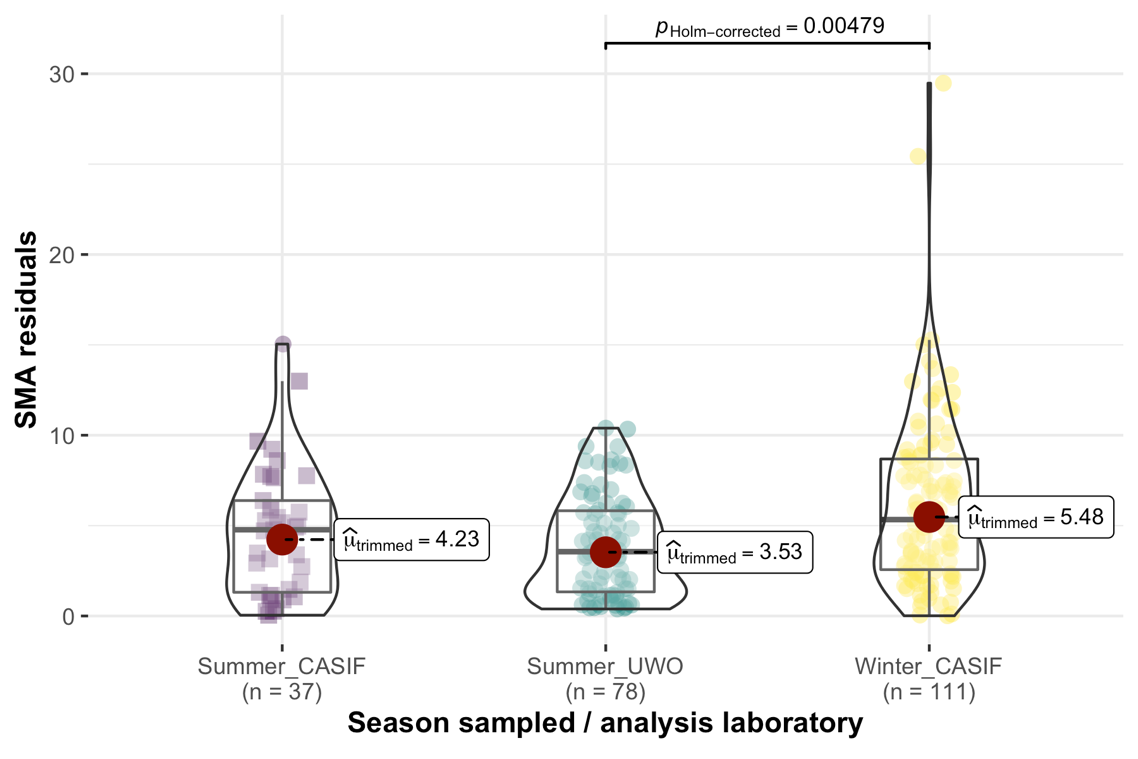
**

Supplemental Figure 2. Residuals from the selected best-fit transfer function, plotted with respect to season of sampling and analysis laboratory. Significant pairwise comparisons are highlighted (*p*_Holm-corrected_ ≤ 0.05).
